# A review of immunotherapy for bone tumors

**DOI:** 10.3389/fimmu.2025.1580598

**Published:** 2025-10-09

**Authors:** Chenwen Wang, Zixiong Chen, Haokun Mo, Jiang Wang, Wei Zhou

**Affiliations:** ^1^ Department of Orthopedics, Liyuan Hospital, Tongji Medical College, Huazhong University of Science and Technology, Wuhan, Hubei, China; ^2^ Department of Orthopedics, Tongji Hospital, Tongji Medical College, Huazhong University of Science and Technology, Wuhan, Hubei, China

**Keywords:** bone tumors, immunotherapy, ICIS, CAR-T cell therapy, oncology vaccines

## Abstract

This article reviews the latest research progress in immunotherapy for bone tumors. Bone tumors are a serious threat to human health, and traditional treatments have limitations. Recently, immunotherapy, as an emerging treatment method, has shown great potential in the treatment of bone tumors. This article systematically introduces the pathological features, traditional treatment methods and limitations of bone tumors, and focuses on the principles, application status and challenges of immune checkpoint inhibitors, CAR-T cell therapy, tumor vaccines and other immunotherapies. At the same time, the combined application strategy of immunotherapy and traditional treatment was discussed, and the future development direction was prospected. The purpose of this article is to provide a reference for the research and clinical application of bone tumor immunotherapy.

## 1 Introduction

A bone tumor is a malignant tumor originating from the skeletal system and can be divided into primary bone tumor and metastatic bone tumor. Primary bone tumors are relatively rare, but they are highly malignant and have a poor prognosis, such as osteosarcoma, which is the most common primary malignant bone tumor, mainly in children and adolescents (1, 2). Osteosarcoma is more likely to occur in the metaphysis of the long bones of the extremities, such as the distal femur, proximal tibia, proximal femur, and proximal humerus (3). Although osteosarcoma has a low incidence, it has high rates of disability and mortality. At present, the standard treatment for osteosarcoma is the neoadjuvant chemotherapy-surgery-consolidation chemotherapy treatment mode, which has achieved great clinical success in patients with localized osteosarcoma (4, 5). Unfortunately, osteosarcoma is a tumor with a high propensity to metastasize, with 15-20% of newly diagnosed osteosarcomas detected for metastasis (6). Metastatic bone tumors are more common and are mostly metastasized from solid tumors such as breast, prostate, and lung cancer. Bone is the most common site of metastasis in breast cancer patients, and 70% of advanced breast cancer cases exhibit bone metastases (7). Due to its large surface area and high vascular supply, bone is a common site for metastatic spread of NSCLC (8, 9). Bone lesions occur in 20-30% of patients with NSCLC at the time of diagnosis, and bone metastases occur in another 35-40% during the course of the disease (10). For patients with metastatic prostate cancer, bone is the primary site of tumor localization and a major cause of disease-related morbidity and mortality (11). Traditional treatments, including surgery, radiotherapy, and chemotherapy, have improved the prognosis of patients to a certain extent but still have limitations such as high recurrence rates and large side effects.

There has been limited progress in survival outcomes in patients with primary or metastatic bone tumors, the prognosis is poor, and the treatment of advanced disease cases is extremely challenging. Traditional chemotherapy drugs do not produce a durable response or cure, and patients may experience severe toxicity (12–14). Due to the limited success of conventional chemotherapy in clinical practice, there is an urgent need to utilize new treatment strategies to improve the treatment of patients with bone tumors, given these bottlenecks in traditional treatment options. In addition to traditional treatments including surgery, chemotherapy and radiotherapy, new treatments such as targeted therapy and immunotherapy are also being studied intensively for bone tumors (15, 16). In recent years, with the rapid development of immunology, immunotherapy, as an emerging anti-tumor treatment, has shown significant efficacy in a variety of solid tumors and hematologic malignancies (17–22). Immunotherapy recognizes and kills tumor cells by activating or enhancing the patient’s own immune system, which has the advantage of strong specificity and relatively few side effects (23). In the field of bone tumors, immunotherapy has also shown great potential, bringing new hope for improving patient outcomes (24).

The purpose of this article is to systematically review the latest research progress in bone tumor immunotherapy, including the application status and challenges of major immunotherapy strategies such as ICIs (immune checkpoint inhibitors), CAR-T cell therapy (chimeric antigen receptor T-cell therapy), and cancer vaccines. At the same time, the combined application strategy of immunotherapy and traditional treatment is discussed, and the future development direction is prospected in order to provide a reference for the research and clinical application of bone tumor immunotherapy.

## 2 Pathological features and traditional treatment methods

Bone tumors can be divided into two categories: benign and malignant, among which malignant bone tumors can be divided into primary osteosarcoma and metastatic bone tumors. Primary osteosarcoma mainly includes osteosarcoma, chondrosarcoma and Ewing sarcoma, which are more common in adolescents and young adults (25). Metastatic bone tumors are more common, mostly metastasized from solid tumors such as breast cancer, prostate cancer, and lung cancer, and are more likely to occur in middle-aged and elderly people (26–28). The pathogenesis of bone tumors is complex, involving a variety of factors such as genetics, epigenetics, and microenvironment (24, 25). Common symptoms include local pain, swelling, pathological fractures, etc. Diagnosis is based on imaging tests (e.g., x-rays, CT, MRI) and histopathological examination (29–32). Traditional treatments include surgery, radiation and chemotherapy (33, 34). Surgery is the mainstay of treatment for bone tumors and aims to completely remove the tumour tissue (35–38); radiotherapy is mainly used for inoperable or postoperative adjuvant therapy; chemotherapy is used for preoperative neoadjuvant therapy and postoperative adjuvant therapy to kill small metastases. Standard treatment for metastatic disease revolves around anthracycline-based chemotherapy, but other drugs including Dacarbazine (39)、Gemcitabine/docetaxel (40)、Ifosfamide (41)、Trabectedin (42)、Pazobanide (43) and Eribulin (44) may be used. However, there are many limitations to traditional treatments. Surgery may lead to limb dysfunction and affect the patient’s quality of life; radiation therapy and chemotherapy can cause serious side effects, such as bone marrow suppression and gastrointestinal reactions. In addition, some patients are insensitive to traditional treatments and are prone to recurrence and metastasis. These limitations have prompted researchers to continuously explore new therapeutic strategies, among which immunotherapy has attracted much attention due to its unique anti-tumor mechanism.

## 3 Immunotherapy fundamentals

Immunotherapy is a therapeutic strategy that recognizes and kills tumor cells by activating or boosting a patient’s own immune system. The basic principle is to use the immune system’s ability to recognize and remove abnormal cells, break the immune escape mechanism of tumors, and restore the body’s immune surveillance and killing function of tumors (45–47). In bone tumor immunotherapy, the following key mechanisms are mainly involved: First, it improves the immune system’s ability to recognize tumor cells by enhancing the presentation of tumor antigens (48); second, tumor-specific T cells are activated and expanded to enhance their killing function (49–51); third, the tumor microenvironment is regulated to overcome immunosuppressive factors (52, 53); Finally, the immune memory function is used to achieve a long-term anti-tumor immune response (54). The advantages of immunotherapy in the treatment of bone tumors are mainly reflected in the following aspects: first, it is highly specific, can accurately target tumor cells, and reduce damage to normal tissues; secondly, it can produce long-lasting immune memory and reduce the risk of recurrence; thirdly, it can work synergistically with traditional treatment methods to improve overall efficacy; Finally, for some refractory or relapsed bone tumors, immunotherapy may offer new treatment options. However, there are also some challenges in immunotherapy, such as large individual differences in efficacy and the possibility of immune-related adverse reactions, which need to be further studied and optimized.

## 4 The main strategy of immunotherapy

Immune checkpoint inhibitors are one of the most widely studied immunotherapy strategies for bone tumors (55). Within tumors, effector T cells have reduced cytokine expression and effector capacity and are resistant to reactivation, a state known as “T cell depletion” (56). Depleted T cells highly express a variety of inhibitory surface molecules that potently prevent T cell activation, including cytotoxic T lymphocyte antigen 4 (CTLA-4), programmed death 1 (PD-1), lymphocyte activation gene-3 (LAG-3), and T cell immunoglobulin and ITIM domain (TIGIT). These inhibitory surface molecules are defined as immune checkpoints (57). PD-1 and its ligand PD-L1 inhibitors and CTLA-4 preparations have shown significant efficacy in a variety of solid tumors (58, 59). Key function of CTLA-4 as a negative regulator of T cell activation. CTLA-4 inhibits further activation of cytotoxic T cells by defeating the binding of the stimulated ligand CD28 to CD80 and CD86 on antigen-presenting cells (APCs), thereby preventing the second signal required for T cell activation (60). The central role of PD-1/PD-L1 in inhibiting T cell-mediated immune responses. PD-L2 also binds to PD-1 and is expressed only on APCs, whereas PD-L1 can be expressed by tumor cells, epithelial cells, dendritic cells, macrophages, and fibroblasts, as well as exhausted T cells. When PD-1 is linked to PD-L1 or PD-L2, downstream TCR signaling and activation are inhibited. PD-L1 expression is upregulated in the presence of interferon γ, possibly originating from tumor-infiltrating effector T cells (61, 62). In the field of bone tumors, studies have shown that the PD-1/PD-L1 pathway is abnormally expressed in tumors such as osteosarcoma and chondrosarcoma, suggesting that it may become a potential therapeutic target (63). Currently, multiple clinical trials are evaluating the efficacy and safety of immune checkpoint inhibitors as monotherapy or in combination with bone tumors. Based on the central role of the OPN-RANKL axis in immune suppression of osseous metastases, several clinical trials have explored the synergy of RANKL inhibitors with ICIs. The Phase Ib/II REVERT trial (NCT04586400) assessed the efficacy of denosumab (anti-RANKL monoclonal antibody) in combination with pembrolizumab in patients with bone metastases in advanced non-small cell lung cancer. Median PFS was 7.9 months in the combination group, significantly better than 4.3 months in the monotherapy group (HR=0.62, P=0.008). More notably, combination therapy increased the ORR for extraosseous lesions from 18% to 42%. The mechanism of action study found that disumab not only inhibited osteoblast activation but also significantly reduced serum OPN levels (an average decrease of 68% after treatment), while increasing the proportion of CD8 + TCF1 + Tpex cells in the tumor (from 3.2% at baseline to 9.8%). This validates the hypothesis in preclinical studies that targeting osteonecrosis cells can reverse systemic immunosuppression (64). In response to the unique metabolic dependence of MTAP-deficient osteosarcoma, researchers proposed an innovative strategy of methionine intervention combined with ICIs. The phase I/II trial of the MAT2A inhibitor SCR6639 (NCT04930081) included 43 patients with MTAP-deficient advanced osteosarcoma. The results showed that the SCR6639 monotherapy group (n = 15) ORR was only 13.3%, and the median PFS was 3.8 months. SCR6639 combined with the pembrolizumab group (n=28), ORR reached 38.5%, and median PFS extended to 7.2 months. Mechanistic studies have shown that MAT2A inhibition increases the expression of PD-L1 by 3–5 times in tumor cells by activating the transcription factor IKZF1 while increasing the secretion of T cell chemoattractants such as CXCL9/10. Tumor biopsies after treatment showed a 4.2-fold increase in the density of CD8^+^ T cell infiltration, which was positively correlated with clinical response (r = 0.78, P < 0.001). A more easily implementable methionine diet restriction program is also being explored. A small-scale pilot study (n=12) in which patients received a daily methionine intake restriction (≤800 mg) combined with nivolumab treatment showed that 50% of patients experienced a metabolic response (PET-CT SUVmax decrease ≥30%), and 3 of them achieved partial remission (65).

CAR-T cell therapy is a type of T cell therapy that genetically engineers T cells to express chimeric antigen receptors (CARs) that specifically recognize tumor antigens, thereby achieving specific killing of tumor cells (66). In 2017, the FDA approved CAR-T cell therapy for the treatment of patients with relapsed or refractory B-acute lymphoblastic leukemia. CAR-T cell therapy involves genetically engineered T cells expressing antigen-specific, non-MHC-restricted receptors that can target and attack specific pathological cells and exert therapeutic effects on patients. The structure of CAR is constantly updated and has now evolved to its fifth generation. In the treatment of bone tumors, investigators are exploring CAR-T cell therapies targeting tumor-associated antigens such as GD2 and HER2 (67, 68). Although CAR-T cell therapy has made breakthroughs in hematologic malignancies (69, 70), the application in solid tumors still faces many challenges, such as immunosuppression and targeted toxicity of the tumor microenvironment. In response to the limitations of traditional CAR-T cells in solid tumors, University College London has developed an innovative OPS-gdT cell platform. The technique uses gamma delta T cells from healthy donors to engineer them to express antibody fragments that target osteosarcoma-related antigens, such as B7-H3, while secreting IL-15 to maintain cell activity. In preclinical osteosarcoma models, OPS-γδT cells showed superior efficacy to conventional CAR-T: Tumor growth inhibition (TGI) was 42% in the CAR-T group; TGI was 89%, and DFS disease-free survival was achieved in 60% of mice treated with OPS-γδT. When combined with a bone sensitizer such as zoledronic acid, TGI is further increased to 97%. Based on these results, we initiated the OPERA-1 trial (NCT05509901), which plans to recruit patients with osteosarcoma to evaluate the safety and preliminary efficacy of OPS-γδT cells (71).

Tumor cells are highly heterogeneous. Therefore, it is important to explore tumor-specific antigens to provide more precise treatments, while oncology vaccines can meet these needs. Oncology vaccines are another important immunotherapy strategy that aims to prevent or treat tumors by activating a patient’s own anti-tumor immune response (72, 73). In the field of bone tumors, researchers are developing vaccines based on tumor-specific antigens, such as NY-ESO-1, MAGE-A3, etc. (74, 75). In addition, personalized neoantigen vaccines have also shown potential application value. However, the research and development of cancer vaccines still faces challenges such as antigen selection and immunogenicity optimization. In the field of advanced neoadjuvant therapy for resectable osteosarcoma, a combination strategy based on dendritic cell (DC) vaccine shows promise. The Phase II NEO-DVIC trial (NCT04201873) compared the efficacy of conventional neoadjuvant chemotherapy with the cDC1 vaccine in combination with pablizumab in patients with primary osteosarcoma: The primary pathological response rate (MPR) of 56% in the cDC1 vaccine group (n = 25) was significantly higher than that of 20% in the chemotherapy group. Median event-free survival (EFS) not reached (vs 15.6 months). Treatment response is strongly associated with CD4+tissue-resident memory T cell (Trm) expansion. Mechanistic studies have shown that the cDC1 vaccine can effectively present tumor antigens to lymph nodes and activate tumor-specific T cell responses, while PD-1 blockade can prevent T cell depletion (76).

Other immunotherapy strategies include oncolytic viruses, immunomodulators, and others. Oncolytic viruses are able to selectively infect and lyse tumor cells while eliciting an anti-tumor immune response (77, 78). In addition to directly lysing tumor cells, the innate immune system can easily recognize the virus as foreign, thus avoiding the need for cancer-specific antigens to initiate an immune response. Oncolytic virus infection can lead to a strong innate immune response through the expression of damage-associated molecular patterns (DAMPs), resulting in local cytokine expression that attracts APCs, natural killer cells, and ultimately T cells (79). To date, four OVs have been clinically approved in select regions for the treatment of various cancers: Rigvir, T-VEC (IMLYGIC), ONYX-015 (DL1520), and H101 (80–83). OV also has some limitations. First, it is possible for the host to produce neutralizing antibodies, and in addition, in the hypoxic tumor core, tumor cells can form necrosis or calcification nearby in response to hypoxia or acidosis, which may limit the efficacy of OVs. Immunomodulators such as interferon, interleukin, etc., can enhance antitumor effects by modulating immune system function. The application of these strategies in the treatment of bone tumors is still in the exploratory stage, and further research is needed to evaluate their safety and efficacy. The suitability of tumor types to major therapies and common adverse reactions are shown in **Table 1**.

**Table 1:** Tumor-type applicability and common adverse effects for major therapies.

Therapy class	Tumor-type applicability	Common adverse effects	Patient selection & biomarkers
Immune Checkpoint Inhibitors (e.g., anti-PD-1/PD-L1, anti-CTLA-4)	Solid tumors: Melanoma, non-small cell lung cancer (NSCLC), renal cell carcinoma, bladder cancer, and many others. Liquid tumors: Hodgkin lymphoma, some primary mediastinal B-cell lymphoma.	Immune-related Adverse Events (irAEs): Can affect any organ. Common ones include colitis (diarrhea), dermatitis (rash), hepatitis, pneumonitis, and endocrinopathies (e.g., thyroiditis).	Selection is often guided by biomarkers such as PD-L1 expression, high Tumor Mutational Burden (TMB), or Microsatellite Instability-High (MSI-H) status, which predict better response.
CAR-T Cell Therapy	Liquid tumors: B-cell acute lymphoblastic leukemia (ALL), certain types of non-Hodgkin lymphoma (e.g., DLBCL), and multiple myeloma. Solid tumors: Largely experimental.	Cytokine Release Syndrome (CRS): Fever, hypotension, and organ dysfunction. Immune Effector Cell-Associated Neurotoxicity Syndrome (ICANS): Confusion, aphasia, seizures. Prolonged cytopenias, increased infection risk.	Selection requires confirmed expression of the target antigen (e.g., CD19 or BCMA). Patients must be fit enough to tolerate severe, acute toxicities.
Oncolytic Viruses	Approved: Melanoma (T-VEC), glioblastoma (Delytact), and head & neck cancer (H101) Clinical Trials: HCC (VG161), pancreatic cancer, bladder cancer, ovarian cancer.	Most common: Flu-like symptoms (fever, chills, fatigue), injection site reactions. Less common: Transient liver enzyme elevations, neuralgia (e.g., facial paralysis). Generally favorable safety profile vs. other immunotherapies.	Tumor susceptibility to viral infection and presence of viral receptors. Predictive biomarkers are emerging (e.g., theViroPredict 1.0 gene signature for VG161 in HCC). May be particularly suited for patients who have failed prior immunotherapies (e.g., PreCPI >3m with HCC showed better OS with VG161).
Cancer Vaccines	Preventive: HPV vaccines for cervical cancer. Therapeutic: - Personalized neoantigen vaccines (PCV): Melanoma, NSCLC, renal cell carcinoma (RCC) - Off-the-shelf: WT1 vaccine for leukemia/ovarian cancer.	Generally mild: Local reactions (injection site redness, swelling, pain), systemic reactions (low-grade fever, fatigue, muscle aches). Rare: Cell cytokine release syndrome (CRS) is uncommon.	Requires identification of immunogenic tumor-specific antigens (e.g., via whole exome/RNA sequencing for PCV). Likely most effective in low tumor burden settings (e.g., adjuvant) and with permissive immune microenvironments. Often combined with checkpoint inhibitors (e.g., mRNA-4157 + pembrolizumab in melanoma).
Bispecific Antibodies (e.g., BiTEs)	Liquid tumors: B-cell ALL (blinatumomab), multiple myeloma.	Toxicities similar to CAR-T but often less severe: CRS, neurotoxicity, and infections.	Requires confirmed target antigen expression (e.g., CD19 for blinatumomab). Often used after prior therapies.

## 5 Challenges and future directions

Although immunotherapy has shown great potential in the treatment of bone tumors, there are still many challenges. First, the immunogenicity of bone tumors is relatively low and highly heterogeneous, resulting in large individual differences in the efficacy of immunotherapy. Second, immunosuppressive factors in the tumor microenvironment, such as regulatory T cells and myeloid-derived suppressor cells, may weaken the efficacy of immunotherapy. In addition, the management of immune-related adverse effects is an important issue. To overcome these challenges, future research directions may include the development of more effective biomarkers to predict immunotherapy response; exploring new immunotherapy targets; optimizing immunotherapy strategies, (such as combining immunotherapy drugs with different mechanisms), specific therapeutic strategies for the bone tumor microenvironment, etc. In addition, strengthening the combination of basic research and clinical translation and carrying out large-scale, multi-center clinical trials are also important directions to promote the development of bone tumor immunotherapy. Another promising direction is the combination of immunotherapy with traditional treatments. For example, radiation and chemotherapy may enhance the efficacy of immunotherapy by inducing immunogenic cell death; immunotherapy prior to surgery may help control micrometastases. The application of OVs combined with ICIs against tumors has shown success in many preclinical studies and has started to become the focus of clinical trials. Exploring the best combination therapy strategy and timing is expected to further improve the treatment effect of bone tumors.

## 6 Conclusion

As an emerging treatment strategy, bone tumor immunotherapy has shown great potential in improving patient outcomes. The application of major immunotherapy strategies such as immune checkpoint inhibitors, CAR-T cell therapy, and tumor vaccines in the treatment of bone tumors is deepening. Although there are still many challenges, with the in-depth understanding of the immune microenvironment and immune escape mechanisms of bone tumors, as well as the development of novel immunotherapy technologies, bone tumor immunotherapy is expected to make breakthroughs in the future. Future research should focus on developing more precise immunotherapy strategies, optimizing combination therapy regimens, and strengthening the translation of basic research and clinical applications. At the same time, large-scale, multicenter clinical trials are needed to evaluate the long-term efficacy and safety of immunotherapy. It is believed that through multidisciplinary collaboration and continuous innovation, immunotherapy will bring new hope to patients with bone tumors and ultimately improve their quality of life and prognosis.
